# Urinoma presenting as an abscess in an immunocompromised host: a case report

**DOI:** 10.1186/1752-1947-7-193

**Published:** 2013-07-26

**Authors:** Rakhee Vaidya, Keith M Swetz

**Affiliations:** 1 Division of Hematology, Mayo Clinic, Rochester, MN, USA; 2 Division of General Internal Medicine, Mayo Clinic, 200 First Street SW, Rochester, MN 55905, USA

**Keywords:** Flank mass, Immunocompromised host, Retroperitoneal space, Urinoma

## Abstract

**Introduction:**

This case report illustrates the relatively rare occurrence of a urinoma masquerading as an abscess in a patient with refractory recurrent non-Hodgkin’s lymphoma. Although urinomas have been reported after urinary instrumentation, our case was confounded by a concurrent pleural effusion, an immunocompromised host, and clinical appearance and subsequent incision and drainage that was consistent with an abscess. Although not the first case reported in the literature, the constellation of findings in our patient demonstrates the importance of including urinoma in the differential diagnosis of patients with flank mass.

**Case presentation:**

Here we report a case of a retroperitoneal fluid collection found in an immunocompromised 65-year-old Caucasian woman. She had recurrent non-Hodgkin’s lymphoma and was neutropenic when the collection was found. Drainage of the fluid demonstrated infection, but the case was complicated by increasing fluid production and the development of an ipsilateral pleural effusion. Despite pleural drainage, the abscess output continued to be high. A computed tomography scan demonstrated fluid collection around the renal calices suggestive of rupture; analysis of the fluid was suggestive of a urinoma. The etiology, pathogenesis, and treatment of urinomas in such patients are discussed.

**Conclusions:**

Urinomas can present in patients who have had urinary tract instrumentation or trauma, but can occur in other hosts. Patients with hematologic malignancy can develop malignant or sympathetic pleural effusions and are at risk for skin and soft tissue infections. Clinicians caring for immunocompromised patients, particularly hematologists, oncologists, and transplant clinicians, should be aware of this potential complication because rapid identification and attempt at correction are important to optimize outcome.

## Introduction

A urinoma is defined as an encapsulated collection of extravasated urine in the perirenal or paraureteral space [1]. It occurs most commonly secondary to trauma to the urinary tract or obstructive uropathy [2]. Prompt diagnosis and correction of the underlying cause is essential in order to prevent complications such as abscess formation and sepsis. Here we present a case of urinoma in an immunocompromised host with non-Hodgkin’s lymphoma presenting with a flank mass that was initially unsuspected due to a confounding ipsilateral pleural effusion.

## Case presentation

A 65-year-old Caucasian woman with refractory non-Hodgkin’s lymphoma and neutropenia was undergoing radiation treatment to a lymphomatous mass involving the left lung apex and adjacent third and fourth ribs. Her past history was also relevant for complicated diverticulitis and a colovaginal fistula requiring surgery 2 years ago. At that time, a small inadvertent ureteral perforation occurred intraoperatively, and an indwelling left ureteral stent was placed and subsequently exchanged every 3 months thereafter. Her urine was persistently colonized with vancomycin-resistant enterococci and *Enterobacter*, for which she was treated multiple times when symptomatic.

**Figure 1:**
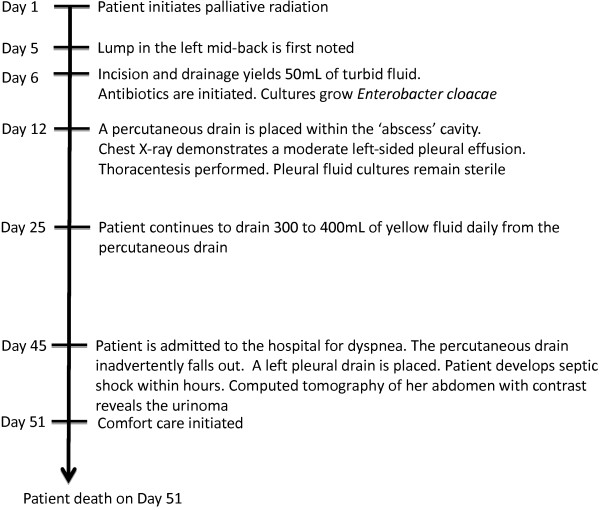
Timeline of clinical events.

During the course of her radiation to the lymphomatous mass (timeline noted in Figure 1), the patient noted a 4×3cm tender fluctuant mass on her left mid-back outside the field of radiation. Laboratory tests showed an absolute neutrophil count of 0.9×10^9^/L, which was consistent with myelosuppression from her most recent systemic chemotherapy. The patient was afebrile and hemodynamically stable, but given her immunocompromised state, immediate surgical consultation was obtained and drainage was recommended. An incision immediately yielded approximately 50mL of turbid fluid, but a drain was unable to be placed at that time. Given the concern of a subcutaneous abscess infected with *Staphylococcus, Streptococcus,* and anaerobic organisms, amoxicillin and clavulanic acid was started initially. A Gram stain demonstrated Gram-negative rods and treatment was continued; however, cultures grew quinolone-sensitive, beta-lactam resistant *Enterobacter cloacae*, and the patient was treated with oral ciprofloxacin for 14 days.

A percutaneous drain was later placed within the ‘abscess’, and over the following 2 weeks, she had increasing drainage (approximately 400mL/day) through it. She also developed a new, moderate-sized left pleural effusion. There was concern that the percutaneous drainage catheter was communicating with the pleural space. Her percutaneous drain inadvertently fell out, and was replaced by a pigtail catheter placed in the pleural space.

**Figure 2:**
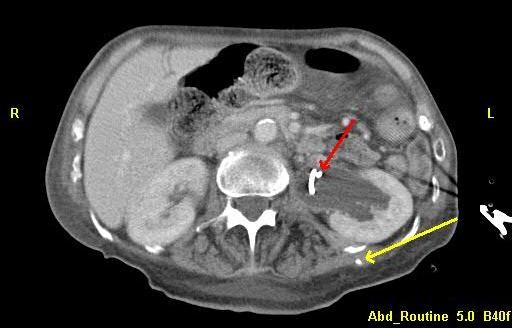
**Computed tomography of the abdomen showing left-sided hydronephrosis with indwelling left ureteral stent (red arrow).** Tip of the percutaneous drain is visualized in the left lateral back (yellow arrow).

**Figure 3:**
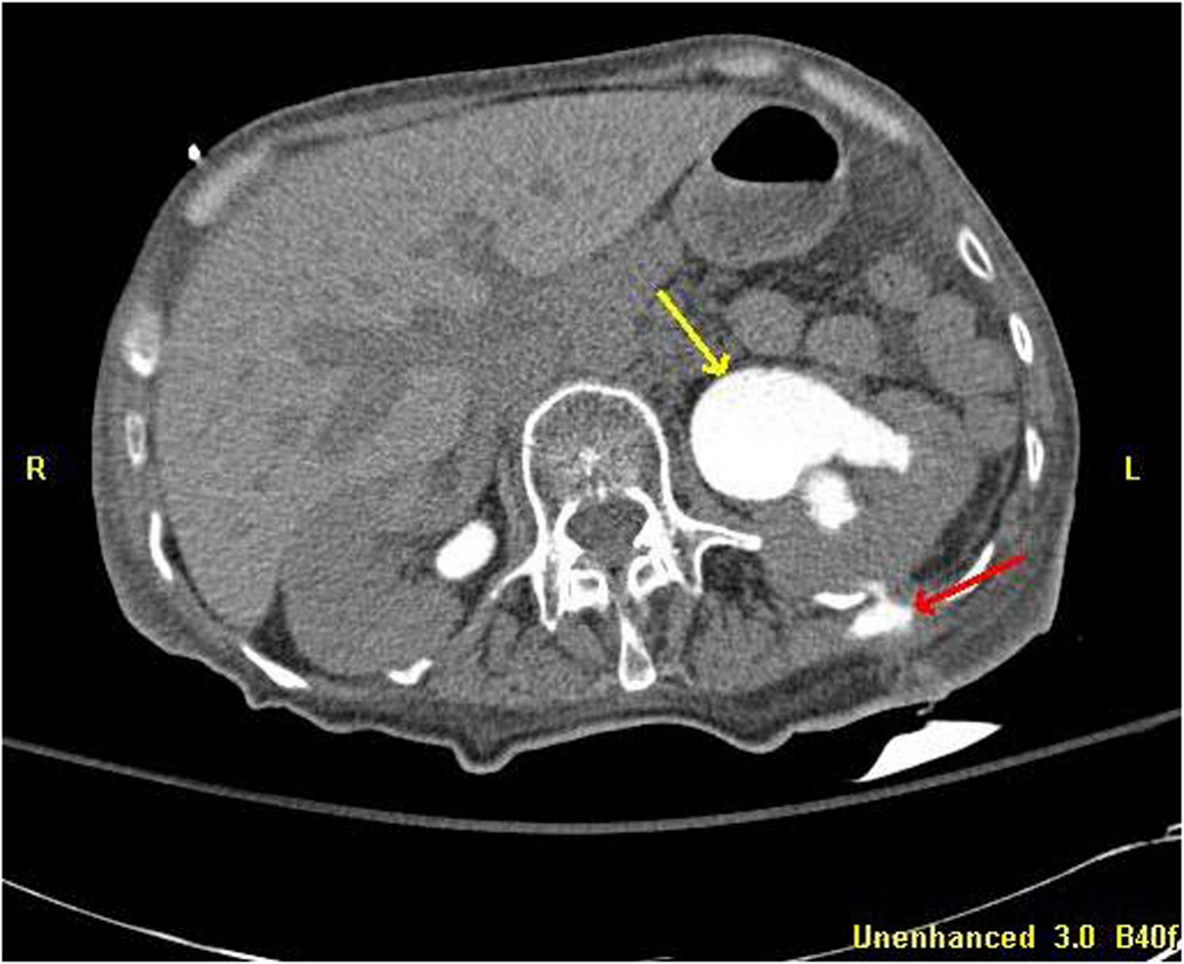
Computed tomography urogram demonstrating contrast extravasation from a calyceal rupture in the mid-left kidney (red arrow) as well as left-sided hydronephrosis (yellow arrow).

Within hours, the patient acutely developed septic shock and was admitted to the intensive care unit. Computed tomography (CT) demonstrated significant left hydronephrosis, suggesting malfunction of the indwelling ureteral stent (Figure 2). A CT urogram demonstrated a ruptured calyx with contrast extravasation at the site of the previously placed percutaneous catheter tip (Figure 3). Creatinine in the ‘abscess’ fluid was elevated at 17.8g/dL. Upon review, our patient’s urine and urinoma grew the same bacteria, whereas the pleural fluid cultures had remained sterile. Given advanced malignancy and sepsis, comfort care was initiated and the patient died.

## Discussion

Our patient presented with an enlarging, tender flank mass that was initially thought to be a subcutaneous abscess. It was treated accordingly with the appropriate antibiotics and prompt percutaneous drainage. The progressively increasing output of straw-colored fluid via the percutaneous catheter along with the concomitant development of an ipsilateral pleural effusion led us to believe that the percutaneous catheter tip was in the pleural space. This led to the placement of a pleural drainage catheter when the percutaneous drain inadvertently fell out. It was only after the patient underwent a CT of her abdomen with intravenous contrast for evaluation of a source of sepsis that a urinoma was revealed. We hypothesize that the falling out of the percutaneous drain probably led to the accumulation of infected urine in her flank which resulted in acute septic shock. Her immunocompromised status and her underlying malignancy were probably significant contributing factors to the rapid development of septic shock that proved fatal for this patient.

A urinoma is an unusual cause of a flank mass, and not typically suspected in the absence of predisposing factors. Urine extravasation in the perinephric space leading to the development of urinoma occurs most commonly after blunt or penetrating renal trauma. Other common causes include transmitted back pressure from an obstructed genitourinary system from ureteral stones, pelvic mass, retroperitoneal fibrosis, or bladder outlet obstruction. In neonates, posterior urethral valves are frequently responsible for the development of hydronephrosis and subsequent urinoma [3]. When suspected, an ultrasound examination is an appropriate screening test to determine the cystic nature of the mass. However, a CT scan is the study of choice in evaluation of masses involving the retroperitoneum [4]. On unenhanced CT, a urinoma usually manifests as a non-specific fluid collection with water attenuation. The attenuation increases progressively after intravenous administration of contrast material because contrast-enhanced urine enters the urinoma [5]. Percutaneous aspiration of the fluid is both diagnostic and therapeutic for small urinomas. Creatinine concentration in a urinoma is much higher than in serum, which helps distinguish them from other retroperitoneal fluid collections [6]. Larger urinomas may require placement of a percutaneous drain, nephrostomy tube or open surgery along with correction of the underlying cause. Untreated urinomas can result in serious complications, including urinary peritonitis, fibrosis, fistulae, abscess formation and septic shock [7].

Our patient had an indwelling ureteral stent that was obstructed despite a regular stent exchange every 3 months. The resultant back pressure caused hydronephrosis and calyceal rupture resulting in a urine leak and urinoma.

## Conclusions

This case illustrates the importance of suspecting urinomas in patients with prior intervention to the urinary tract who present with a flank mass or fluid collection. An immunocompromised host may present further challenges because classic symptoms of pain or leukocytosis from infection may be masked.

## Consent

Written informed consent was obtained from the patient’s surrogate decision-maker/next-of-kin for publication of this case report and accompanying images. A copy of the written consent is available for review by the Editor-in-Chief of this journal.

## Competing interests

The authors declare that they have no competing interests.

## Authors’ contributions

RV analyzed and interpreted the patient data regarding the hematological disease and its complication. KMS analyzed and interpreted the patient data and was a major contributor in writing the manuscript. All authors read and approved the final manuscript.
